# *In vitro* activity of ceftazidime-avibactam, imipenem-relebactam, aztreonam-avibactam, and comparators toward carbapenem-resistant and hypervirulent *Klebsiella pneumoniae* isolates

**DOI:** 10.1128/spectrum.02806-23

**Published:** 2023-11-20

**Authors:** Dan Li, Hua Yu, Xiangning Huang, Shanshan Long, Jie Zhang

**Affiliations:** 1 School of Medicine, University of Electronic Science and Technology of China, Chengdu, China; 2 Department of Laboratory Medicine, Sichuan Provincial Key Laboratory for Human Disease Gene Study and the Center for Medical Genetics, Sichuan Academy of Medical Sciences and Sichuan Provincial People's Hospital, University of Electronic Science and Technology of China, Chengdu, Sichuan, China; 3 Department of Laboratory Medicine, Medical Center Hospital of Qionglai City, Chengdu, Sichuan, China; University of Saskatchewan, Saskatoon, Saskatchewan, Canada

**Keywords:** hypervirulent *Klebsiella pneumoniae*, ceftazidime-avibactam, imipenem-relebactam, carbapenem-resistant, aztreonam-avibactam

## Abstract

Carbapenem-resistant and hypervirulent *Klebsiella pneumoniae* (CR-hvKP) strains are increasingly reported, posing a significant threat to public health. Therefore, effective antimicrobial therapy is urgently needed. This study aimed to analyze the *in vitro* activity of ceftazidime-avibactam (CZA), imipenem-relebactam (IMR), and aztreonam-avibactam (AZA) toward CR-hvKP and carbapenem-resistant *Klebsiella pneumoniae* (CRKP) isolates. Non-repetitive clinical CRKP and CR-hvKP strains were collected from Sichuan Provincial People’s Hospital between August 2018 and June 2022. CR-hvKP strains were screened using string tests and polymerase chain reaction (PCR). The microbroth dilution method was used to evaluate *in vitro* antibacterial activity of CZA, IMR, and AZA toward CRKP and CR-hvKP stains. The molecular characteristics of CRKP and CR-hvKP strains were investigated using PCR amplification. The virulence features of CR-hvKP strains were investigated using serum resistance assays and a *Galleria mellonella* infection model. A total of 114 CRKP and 40 CR-hvKP strains were collected. The susceptibility rates of CRKP and CR-hvKP to tigecycline, colistin, and polymyxin B exceeded 89.5%. The susceptibility rates of CRKP and CR-hvKP to CZA were 64.0% and 77.5%, respectively; the susceptibility rates to IMR were 92.5% and 71.9%, respectively; and the susceptibility rates to AZA were 89.5% and 75.0%, respectively. Multilocus sequence typing and *wzi*-loci sequencing identified sequence type 11 KL64 as the predominant type in CRKP and CR-hvKP strains. Carbapenemase genes were dominated by *bla*KPC-2 and *bla*NDM-1. IMR and AZA may be promising therapeutic agents for the treatment of infections caused by CRKP and CR-hvKP isolates.

**IMPORTANCE:**

To our knowledge, this is the first study to report the *in vitro* activity of two novel antimicrobial drugs, including imipenem-relebactam (IMR) and aztreonam-avibactam (AZA), toward carbapenem-resistant and hypervirulent *Klebsiella pneumoniae* (CR-hvKP) strains. Our *in vitro* activity study revealed that only few antibacterial agents (including several novel agents) exhibit high antimicrobial activity toward carbapenem-resistant *Klebsiella pneumoniae* (CRKP) and CR-hvKP isolates. IMR and AZA may be promising therapeutic agents for the treatment of infections caused by CRKP and CR-hvKP isolates.

## INTRODUCTION

*Klebsiella pneumoniae* (KP), which belongs to *Enterobacteriaceae*, is one of the most threatening pathogens and an important source of antimicrobial resistance (1). Currently, there are two circulating pathotypes, namely hypervirulent *KP* (hvKP) and classical *KP* (cKP) (2). Clinically, cKP presents a challenge owing to its ability to acquire antimicrobial resistance elements, whereas hvKP isolates remain susceptible to most antimicrobial agents but exhibit greater virulence than cKP, primarily causing community-acquired infections (2, 3). Although cKP and hvKP have evolved in a parallel manner over the past few decades, carbapenem-resistant and hypervirulent *KP* (CR-hvKP) strains are increasingly reported (4 – 6). These strains have both antimicrobial resistance determinants and virulence factors on the same plasmids, posing an even greater clinical challenge.

Over the past few years, several novel antimicrobial agents, including plazomicin, cefiderocol, eravacycline, and β-lactam/β-lactamase inhibitor combinations, such as ceftazidime-avibactam (CZA) and imipenem-relebactam (IMR), were approved by the United States Food and Drug Administration (FDA) for the treatment of complicated intraperitoneal infections and complicated urinary tract infections (7). Unfortunately, these β-lactam/β-lactamase inhibitor combinations are primarily used to inhibit serine β-lactamases-producing carbapenem-resistant *Enterobacteriaceae* (CRE) but not metallo-β-lactamases. In contrast, aztreonam (ATM) is the only monocyclic β-lactam that is not hydrolyzed by metallo-β-lactamases. Aztreonam-avibactam (AZA), another novel β-lactam/β-lactamase inhibitor combination, can, therefore, simultaneously target different carbapenemases, and may be used to treat infections caused by carbapenem-resistant *Klebsiella pneumoniae* (CRKP) and CR-hvKP strains that produce different types of carbapenemases (8). AZA is currently undergoing clinical trials pending regulatory approval (NCT03580044) (8). New antimicrobial agents offer hope for the clinical treatment of CRKP and CR-hvKP infections, which exhibit a concerning epidemic trend in China. However, the antibacterial activity of these new antibacterial drugs toward CR-hvKP *in vitro* has not been reported. We therefore investigated the *in vitro* antibacterial activity of some of these new drugs toward CRKP and CR-hvKP to provide a basis for the clinical treatment of CRKP and CR-hvKP infections.

## MATERIALS AND METHODS

### Bacterial isolates

Between August 2018 and June 2022, a total of 2,529 KP strains were collected from Sichuan Provincial People’s Hospital. Among them, 114 CRKP and 40 non-repetitive CR-hvKP isolates were identified. The isolates were obtained from different samples, including sputum, urine, blood, and pus. In this study, CRKP was defined as clinical KP strains that are resistant to at least one carbapenem [ertapenem, imipenem (IPM), meropenem (MEM), or doripenem] according to the 2020 Clinical Laboratory Standards Institute (CLSI) breakpoints(9, 10); hvKP was defined as a positive string test (>5 mm) and co-harboring the genes *rmpA/rmpA2* and *iucA*, as previously described (11). We used PCR and subsequent Sanger sequencing to detect virulence genes. The virulence gene primers are listed in Table S1.

### Tests for antibiotic sensitivity

A VITEK-2 compact system (bioMérieux, France) was used to determine strain identity, and matrix-assisted laser desorption/ionization mass spectrometry (Ci-phergen Biosystem, USA) was used to confirm the identity of all strains. A MIC broth microdilution method (Thermo Scientific Sensititre AIM Automated Inoculation Delivery System, USA) was used to determine antibiotic sensitivity. The MICs of IPM, IMR,MEM, ceftazidime (CAZ), CZA, colistin (COL), polymyxin B (POL), amikacin (AMK), cefepime (FEP), ATM, AZA, ciprofloxacin (CIP), tigecycline (TGC), and trimethoprim-sulfamethoxazole (SXT) were determined in accordance with CLSI breakpoints (2020 version) (9). The MICs of TGC and AZA were determined according to FDA (12) criteria and European Committee on Antimicrobial Susceptibility Testing (EUCAST) (13) breakpoints, respectively. *KP* ATCC 700603, *Escherichia coli* ATCC 25922, and *Pseudomonas aeruginosa* ATCC 27853 served as quality control strains.

### String tests

String tests were performed to identify the hypermucoviscous phenotype as previously described (4). All CRKP and CR-hvKP isolates were inoculated on 5% sheep blood agar plates and incubated at 37°C overnight. The string test was considered positive upon the formation of a viscous string greater than 5 mm in length.

### Antibiotic resistance genes and capsular serotyping

We used PCR analyses and subsequent Sanger sequencing to detect carbapenemase genes (*bla*KPC, *bla*NDM, *bla*VIM, *bla*IMP, *bla*SME, *bla*GES, and *bla*OXA-48-like), as well as other resistance genes (*bla*CTX-M-1, *bla*CTX-M-2, *bla*CTX-M-8, *bla*CTX-M-9 *bla*SHV, and *bla*TEM). The boiling method was used to extract the genomic DNA of KP. The PCR products were visualized on a 1.5% agarose gel. Similarly, PCR analysis was used to detect the capsular type of KP, followed by *wzi-*loci sequencing, as previously described (14). The specific primers used in this section are listed in Tables S2 and S3.

### Multilocus sequence typing

All CRKP strains, including CR-hvKP, were genotyped by multilocus sequence typing (MLST) (15). Seven housekeeping genes (*gapA, infB, mdh, phoE, pgi, rpoB,* and *tonB*) were detected by PCR, followed by Sanger sequencing, in accordance with the protocol on the MLST website (16). The primers used in this study are listed in Table S4.

### Serum resistance assay

Serum resistance assays were conducted by mixing CR-hvKP with human serum obtained from 10 healthy volunteers as previously described (4, 17, 18). Briefly, 100 µL CR-hvKP strains in mid-log phase were mixed to a final concentration of 1 × 10⁶ CR-hvKP CFU/mL with 300 µL human serum in a 1:3 ratio and incubated at 37°C. At 0, 1, 2, and 3 h, 100 µL aliquots were plated on Luria broth agar and incubated at 37°C overnight to determine the number of CFUs. All assays were performed in triplicate.

### *Galleria mellonella* infection models

Among the 40 CR-hvKP strains, 10 strains were randomly selected to verify *in vivo* virulence in *Galleria mellonella*. Eight randomly selected *G. mellonella* larvae, weighing approximately 350 mg (Tianjin Huiyude Biotech Company), were selected for each isolate and maintained on woodchips in the dark at approximately 15°C. The CR-hvKP concentration was adjusted to 1 × 10^7^ CFU/mL with PBS, and a 10-µL aliquot was administered via the rear left proleg to infect the *G. mellonella*, as previously described (4). The survival rates of *G. mellonella* were recorded every 12 h. The hypervirulent strain SC43-25, isolated from blood samples of a 65-year-old human admitted for liver abscess and subsequent bloodstream infection and endophthalmitis, was selected as the positive virulence control. Whole genome sequence analysis revealed that this strain (GenBank JAVKOU000000000) is highly homologous to the typical hypervirulent strain NTUH-K2044. The classic ST11 strain SC12-22 (GenBank JAVJNH000000000) (string test/*iucA/rmpA/rmpA2* negative) was selected as the negative virulence control. These experiments were conducted in duplicate.

## RESULTS

### Isolate identification

A total of 154 CRKP strains were collected, of which 40 CR-hvKP strains were identified by string tests and PCR amplification of the *rmpA/rmpA2* and *iucA* genes.

### Antimicrobial susceptibility

The antimicrobial susceptibility results are summarized in Table 1. In this study, the CRKP and CR-hvKP strains exhibited low (0.0%–14.0%) susceptibility rates to IPM, MEM, CAZ, FEP, CIP, and ATM, and variable susceptibility rates to the novel antibacterial agents. The CR-hvKP group exhibited greater susceptibility rates to IMR (92.5%) than the CRKP group (71.9%). Of the CRKP strains, 89.5% exhibited susceptibility to AZA compared with 75.0% of the CR-hvKP strains. In addition, the susceptibility rates of CRKP and CR-hvKP to CZA were 64.0% and 77.5%, respectively. Both groups exhibited the highest susceptibility rates to COL, POL, and TGC (>89.5%).

**TABLE 1:** Antimicrobial susceptibility of CRKP and CR-hvKP strains^*a*^

Antimicrobial agents	CR*KP* (*n* = 114)			CR-hv*KP* (*n* = 40)		
	Susceptibility rates (%)	MIC_50_	MIC_90_	Susceptibility rates (%)	MIC_50_	MIC_90_
IPM	4.4	64	128	12.5	64	128
IMR	71.9	1	64	92.5	1	2
MEM	1.8	256	256	7.5	256	256
CAZ	0.0	256	256	2.5	256	256
CZA	64.0	4	256	77.5	8	16
COL	89.5	1	4	92.5	1	1
POL	90.4	0.5	2	92.5	0.5	0.5
AMK	49.1	128	256	25.0	256	256
FEP	2.6	256	256	5.0	256	256
ATM	9.6	256	256	5.0	256	256
CIP	14.0	128	256	10.0	128	256
SXT	45.6	4	256	40.0	256	256
AZA	89.5	1	4	75.0	2	4
TGC	95.6	1	4	92.5	2	4

^*a*^ IPM, Imipenem; IMR, Imipenem-relebactam; MEM, Meropenem; CAZ, Ceftazidime; CZA, Ceftazidime-Avibactam; COL, Colistin; POL, Polymyxin B; AMK, Amikacin; FEP, Cefepime; ATM, Aztreonam; CIP, Ciprofloxacin; SXT, Trimethoprim-sulfamethoxazole; AZA, Aztreonam-avibactam; TGC, Tigecycline.

### Resistance genes

The distribution of drug resistance genes is summarized in Table 2. In the CRKP strains, *bla*KPC-2 was the predominant carbapenemase gene (67/114, 58.8%), followed by *bla*NDM-5 (17/114, 14.9%) and *bla*NDM-1 (9/114, 7.9%). Among the CR-hvKP strains, 82.5% harbored *bla*KPC-2 and 7.5% harbored *bla*NDM-1. The *bla*SME, *bla*GES, and *bla*OXA-48 genes were not detected in the CRKP or CR-hvKP strains. *bla*SHV was harbored by 98.2% (112/114) of the CRKP and 97.5% (39/40) of the CR-hvKP isolates, whereas *bla*TEM was harbored by 36.8% (42/114) of the CRKP and 37.5% (15/40) of the CR-hvKP isolates. The detection rates of *bla*CTX-M in CRKP and CR-hvKP isolates were 71.1% and 80.0%, respectively.

**TABLE 2:** Resistance genes of CRKP and CR-hvKP strains

Resistance gene	CR*KP* (*N* = 114)		CR-hv*KP* (*N* = 40)	
	*N*	%	*N*	%
*bla*_KPC-2_	67	58.8	33	82.5
*bla*_NDM-1_	9	7.9	3	7.5
*bla*_NDM-5_	17	14.9	0	0.0
*bla*_NDM-2_	1	0.9	0	0.0
*bla*_KPC-3_	2	1.8	1	2.5
*bla*_KPC-9_	1	0.9	0	0.0
*bla*_KPC-14_	1	0.9	0	0.0
*bla*_IMP-38_	1	0.9	0	0.0
No carbapenemase detected	15	13.2	3	7.5
*bla*_SHV_	112	98.2	39	97.5
*bla*_TEM_	42	36.8	15	37.5
*bla*_CTX-M_	81	71.1	32	80.0

### Capsular serotyping

The distribution of capsular serotyping in CRKP and CR-hvKP was determined using *wzi*-typing (Table 3). Of the 114 CRKP isolates, PCR and Sanger sequencing revealed that 49.1% (56/114) were capsular type KL64, followed by capsular types KL47 (10/114, 8.8%), KL54 (5/114, 4.4%), and KL18 (5/114, 4.4%). Of the CR-hvKP isolates, 82.5% (33/40) were capsular type KL64, followed by capsular types KL1 (2/40, 5.0%), KL2 (2/40, 5.0%), KL115 (1/40, 2.5%), KL62 (1/40, 2.5%), and KL54 (1/40, 2.5%).

**TABLE 3:** Capsular serotyping of CRKP and CR-hvKP strains

Capsular serotyping	CR*KP* (*N* = 114)		CR-hv*KP* (*N* = 40)	
	*N*	%	*N*	%
KL1	0	0.0	2	5.0
KL2	0	0.0	2	5.0
KL10	2	1.8	0	0.0
KL18	5	4.4	0	0.0
KL19	2	1.8	0	0.0
KL25	3	2.6	0	0.0
KL28	3	2.6	0	0.0
KL31	2	1.8	0	0.0
KL47	10	8.8	0	0.0
KL54	5	4.4	1	2.5
KL62	1	0.9	1	2.5
KL64	56	49.1	33	82.5
KL81	2	1.8	0	0.0
KL102	3	2.6	0	0.0
KL106	2	1.8	0	0.0
KL115	2	1.8	1	2.5
Others	16	14.0	0	0.0

### MLST type

The distribution of MLST typing in CRKP and CR-hvKP is summarized in Table 4. MLST revealed that 58.8% of the CRKP isolates belong to ST11 (67/114), followed by ST789 (5/114, 4.4%), ST15 (5/114, 4.4%), and ST29 (4/114, 3.5%). Meanwhile, 80.0% of the CR-hvKP isolates belong to ST11 (32/40); the other STs were ST29 (1/40, 2.5%), ST23 (1/40, 2.5%), ST65 (1/40, 2.5%), ST367 (1/40, 2.5%), and ST3176 (1/40, 2.5%).

**TABLE 4:** MLST of CRKP and CR-hvKP strains

MLST	CR*KP* (*N* = 114)		CR-hv*KP* (*N* = 40)	
	*N*	%	*N*	%
ST11	67	58.8	32	80.0
ST15	5	4.4	0	0.0
ST17	2	1.8	0	0.0
ST23	0	0.0	1	2.5
ST29	4	3.5	1	2.5
ST65	0	0.0	1	2.5
ST101	3	2.6	0	0.0
ST147	2	1.8	0	0.0
ST307	2	1.8	0	0.0
ST367	0	0.0	1	2.5
ST485	2	1.8	0	0.0
ST789	5	4.4	0	0.0
ST3176	0	0.0	1	2.5
Others	22	19.3	4	10.0

### Serum resistance and *G. mellonella* infection model

**Fig 1:**
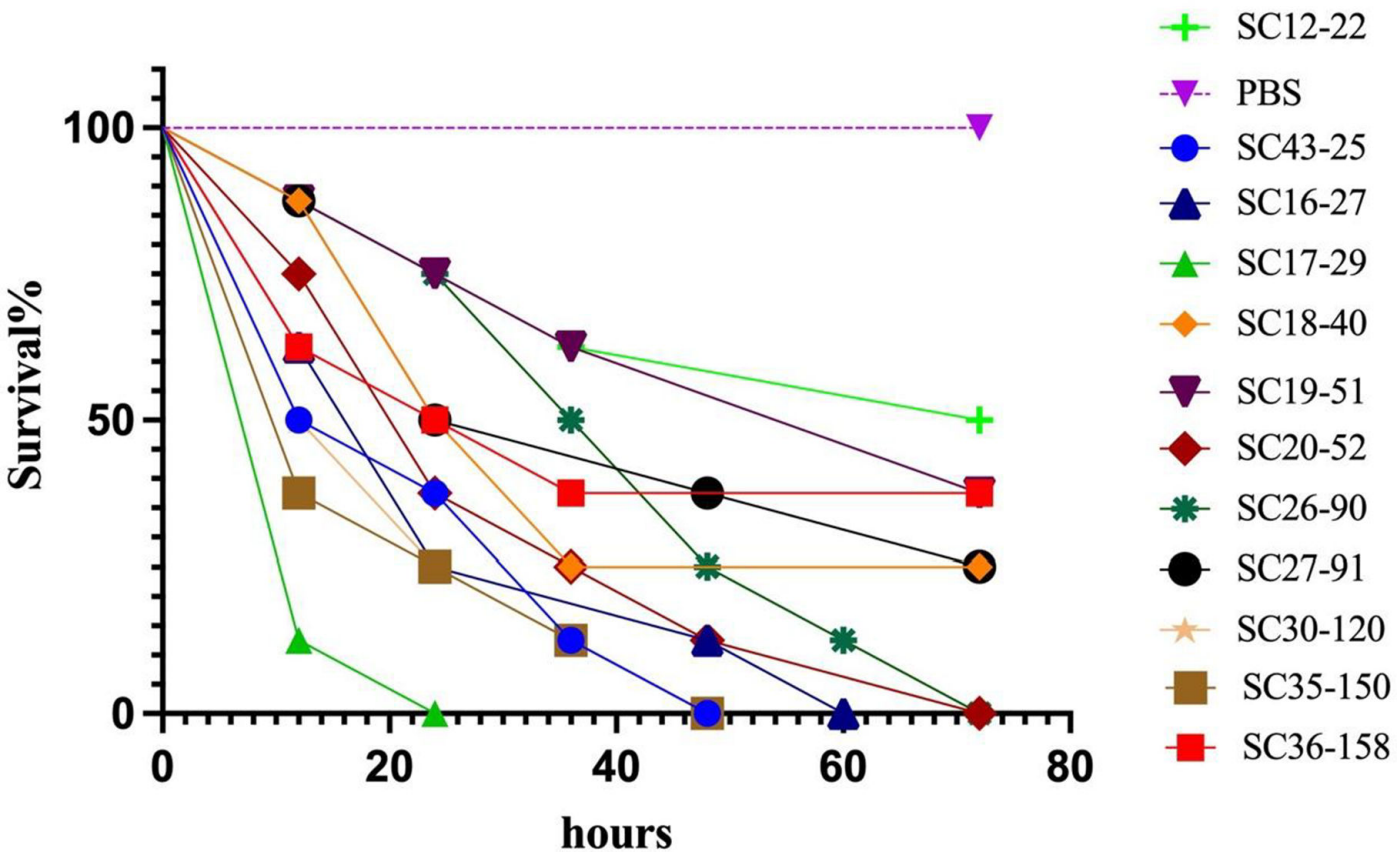
Virulence potential of CR-hvKP strains in a *Galleria mellonella* infection model.

Of the 40 CR-hvKP strains tested for serum resistance, 27 exhibited insensitivity (grade 5 or 6), 10 exhibited moderate sensitivity (grade 3 or 4), and 3 exhibited high sensitivity (grade 1 or 2). The virulence potential of the CR-hvKP strains in a *G. mellonella* infection model is shown in Fig. 1 and Table 5. In our study, all PBS-injected control insects survived for 72 h; none of the positive control insects (Control, infected with SC43-25) survived at 48 h (median survival time, 24 h); and negative control insects (negative control, infected with the classic ST11 KL64 strain SC12-22) exhibited 50% survival at 72 h. Of the 10 tested CR-hvKP strains, SC17-29 (ST11, KL64) was the most virulent strain, with all larvae dying within 24 h (median survival time, 12 h), followed by ST86 KL2 SC30-120 (0% survival at 72 h; median survival time, 18 h), and ST23 KL1 SC35-150 (0% survival at 48 h; median survival time, 12 h). All 10 tested CR-hvKP strains led to shorter median survival times than the negative control, ST11 KL64 strain SC12-22.

**TABLE 5:** Characteristics of 10 selected CR-hvKP strains

Sample	SC43-25 (positive)	SC12-22 (negative)	SC16-27	SC17-29	SC18-40	SC19-51	SC20-52	SC26-90	SC27-91	SC30-120	SC35-150	SC36-158
Median survival (hours)	18	72	24	12	30	72	24	42	36	18	12	30
ST	23	11	11	11	11	367	29	889	65	86	23	11
KL	KL1	KL64	KL64	KL64	KL64	KL1	KL62	KL54	KL2	KL2	KL1	KL64
Carbapenemase	-	KPC-2	KPC-2	KPC-2	-	NDM-1	NDM-1	NDM-1	-	KPC-2	-	KPC-2

## DISCUSSION

To our knowledge, this is the first study to report the *in vitro* activity of two novel antimicrobial drugs, including IMR and AZA, toward CR-hvKP strains.

Antimicrobial resistance has become a major global public health challenge, with nearly 5 million infections worldwide in 2019, resulting in at least 1.27 million deaths (19). CRE, most commonly CRKP, plays an important role in the emergence of antimicrobial resistance, and there are limited treatment options for infections caused by CRKP and CR-hvKP. In our study, all CRKP and CR-hvKP strains exhibited low susceptibility to IPM, MEM, CAZ, FEP, ATM, AMK, and SXT, which is consistent with previous studies (20, 21). TGC, COL, and POL exhibited greater antibacterial activity toward both CRKP and CR-hvKP, with susceptibility rates exceeding 89.5%, indicating that they may maintain a high level of activity against these two bacteria. However, the toxic side effects of COL and POL limit their clinical use (22). TGC, a glycylcycline peptide antibacterial drug derived from minocycline, inhibits bacterial protein synthesis and is effective against a wide range of bacteria, including CRKP (23). Its activity is independent of carbapenemase presence and type. However, TGC use is generally limited to the treatment of intra-abdominal infections as rapid distribution in tissues after administration limits serum and urinary concentrations. High doses of TGC may be more effective than standard TGC doses for treating complex intra-abdominal infections (24). In recent years, the availability of several novel β-lactam/β-lactamase inhibitor combinations has given hope for the clinical treatment of CRKP. In this study, drug susceptibility tests were performed for IMR, CZA, and AZA. Among them, CZA, marketed in China since 2019, is mainly used to treat infections caused by CRE. This study showed that the susceptibility rates of CRKP and CR-hvKP to CZA were 64.0% and 77.5%, respectively, which are lower than the 92.5% reported by Han Jing (25) and 81.0% reported by Huang Liping (26), respectively. The difference in the susceptibility rates of CRKP and CR-hvKP to CZA may be related to different types of carbapenemase produced by each group. For example, we found that 58.8% of CRKP strains harbored *bla*KPC-2, whereas 82.5% of CR-hvKP strains harbored *bla*KPC-2. Only CRKP strains harbored *bla*NDM-5 (14.9%). Although IMR, CZA, and meropenem/vaborbactam are considered the treatment of choice for extra-urinary KPC-producing CRE infections (24), IMR is not yet available in China. The antibacterial activity of IMR appears to be higher toward CR-hvKP than CRKP (susceptibility rates of 92.5% and 79.1%, respectively), suggesting that IMR may hold promise for the treatment of CR-hvKP infection, but additional molecular and clinical data are required. As AZA may simultaneously target different types of carbapenemases, it could theoretically be used to treat CRKP and CR-hvKP infections that produce variable carbapenemases. This study showed that the susceptibility rates of CRKP and CR-hvKP to AZA were 89.5% (MIC_50_ = 1 mg/L, MIC_90_ = 4 mg/L) and 75.0% (MIC_50_ = 2 mg/L, MIC_90_ = 4 mg/L), respectively (with a breakpoint referenced to the EUCAST for ATM (8), suggesting that AZA may maintain high activity toward CRKP. However, only 75% of CR-hvKP strains retained susceptibility to AZA (MIC_50_ = 2 mg/L, MIC_90_ = 4 mg/L) even before clinical use. These susceptibility rates are much lower than those recently reported by Vázquez-Ucha et al. (98.2%, MIC_50_ ≤ 1 mg/L, MIC_90_ ≤ 1 mg/L) (8). The mechanisms of AZA resistance remain largely unknown, and additional studies are needed to investigate the resistance mechanisms and molecular characteristics to curb the spread and development of this drug-resistant bacteria.

Comparisons between CRKP and CR-hvKP imply that hypervirulence may affect antibiotic susceptibility. However, this could not be validated in this study since the resistance backgrounds of the isolates vary greatly. In China and in Sichuan Provincial People’s Hospital, KPC-2-producing ST11 KL64 is the most common type among CR-hvKP strains (27). Therefore, the main putative evolutionary path of CR-hvKP may be a classic KPC-2-producing ST11 KL64 CRKP strain acquiring a virulence plasmid. However, whether this results in a fitness cost, such as a decreased MIC for carbapenems, remains unknown. One study reported by Zhang et al. (27) demonstrated that there was limited fitness cost for three KPC-2-producing ST11 CR-hvKP isolates compared to CRKP. Another study (28) reported that ST23-K1 hvKP isolates might compromise virulence and demonstrate a lower fitness cost. To our knowledge, there is no relevant literature report on the fitness cost of CRKP acquiring a pLVPK-like plasmid. Further studies are needed to evaluate whether acquiring a virulence phenotype influences the antimicrobial susceptibility of a classic CRKP strain.

This study has several limitations. Firstly, the identification of CR-hvKP included strains with positive string tests and *rmpA/rmpA2* and *iucA* genes. However, not all hvKP isolates are string-test positive (29), and our results may, therefore, have underestimated the incidence of CR-hvKP. Secondly, delineation of hvKP virulence genes remains incomplete (29), and the combination of virulence genes required for maximal virulence remains unknown. The biomarkers for hypervirulence used in this study (*rmpA/rmpA2* and *iucA* genes), therefore, do not guarantee hypervirulence, and additional animal model studies are necessary. Thirdly, this was a single-center retrospective study, and clinical features and outcomes were not presented.

In conclusion, our *in vitro* activity study revealed that only few antibacterial agents, including several novel agents, exhibit high antimicrobial activity toward CRKP and CR-hvKP isolates. Among these, two novel β-lactam/β-lactamase inhibitor combinations—IMR and AZA—may be promising therapeutic agents for treating infections caused by CRKP and CR-hvKP isolates, especially KPC-2-producing ST11 KL64, and further clinical evaluation of the efficacy of these agents is warranted.

## Data Availability

The whole genome sequences of the two strains (SC43-25 and SC12-22) in this study were deposited in the GenBank database (accession no. JAVKOU000000000 and JAVJNH000000000, respectively).

## SUPPLEMENTAL MATERIAL

Tables S1 to S4 spectrum.02806-23-s0001.docx Primers used in this study.

ASM does not own the copyrights to Supplemental Material that may be linked to, or accessed through, an article. The authors have granted ASM a non-exclusive, world-wide license to publish the Supplemental Material files. Please contact the corresponding author directly for reuse.
