# Supplementary material for: In vitro activity of ceftazidime-avibactam, imipenem-relebactam, aztreonam-avibactam, and comparators toward carbapenem-resistant and hypervirulent Klebsiella pneumoniae isolates
Source: Microbiol Spectr. 2023 Nov 20;11(6):e02806-23. doi: 10.1128/spectrum.02806-23 (PMC10848889; doi:10.1128/spectrum.02806-23)
Supplement: Tables S1 to S4 — Primers used in this study. [file spectrum.02806-23-s0001.docx]

**Supplementary Materials**

Table S1 Primers of virulence genes of KP

| Virulence genes | Primers（5’→ 3’） | Product (bp) | Temperature (℃) |
| --- | --- | --- | --- |
| *rmpA* | F：ACTGGGCTACCTCTGCTTCA  R：CTTGCATGAGCCATCTTTCA | 516 | 58 |
| *rmpA2* | F：TGTGCAATAAGGATGTTACATTAGT  R：TTTGATGTGCACCATTTTTCA | 609 | 56 |
| *iucA* | F：GCTTATTTCTCCCCAACCC  R：TCAGCCCTTTAGCGACAAG | 583 | 59 |

Table S2 Primers of resistance genes of KP

| Resistance genes | Primers（5’ → 3’） | Product (bp) | Temperature (℃) |
| --- | --- | --- | --- |
| *bla*_KPC_ | F: ATGTCACTGTATCGCCGT C  R: TTACTGCCCGTTGACGCC | 882 | 59 |
| *bla*_SME_ | F: AACGGCTTCATTTTTGTTTAG  R: GCTTCCGCAATAGTTTAG | 830 | 58 |
| *bla*_GES_ | F: ATGCGCTTCATTCACGCAC  R: CTATTTGTCCGTGCTCAGG | 846 | 62 |
| *bla*_VIM_ | F: GCMCTTCTCGCGGAGATTGA  R: TGCGCAGCACCRGGATAGA | 257 | 59 |
| *bla*_NDM_ | F: GAAGCTGAGCACCGCATTAG  R: GGGCCGTATGAGTGATTGC | 982 | 58 |
| *bla*_OXA-48_ | F: TTGGTGGCATCGATTATCGG  R: GAGCACTTCTTTTGTGATGGC | 743 | 62 |
| *bla*_IMP_ | F: CTACCGCAGCAGAGTCTTTG  R: AACCAGTTTTGCCTTACCAT | 587 | 55 |
| *bla*_TEM_ | F: ATAAAATTCTTGAAGACGAAA  R: GACAGTTACCAATGCTTAATCA | 1080 | 58 |
| *bla*_SHV_ | F: CGCCGGGTTATTCTTATTTGTCGC  R: TCTTTCCGATGCCGCCGCCAGTCA | 1017 | 68 |
| *bla*_CTX-M-1_ | F: CAGCGCTTTTGCCGTCTAAGC  R: GGCCCATGGTTAAAAAATCACTGC | 945 | 62 |
| *bla*_CTX-M-2_ | F: CTCAGAGCATTCGCCGCTCA  R: CCGCCGCAGCCAGAATATCC | 848 | 59 |
| *bla*_CTX-M-8_ | F: ACTTCAGCCACACGGATTCA  R: CGAGTACGTCACGACGACTT | 1024 | 848 |
| *bla*_CTX-M-9_ | F: GTTACAGCCCTTCGGCGATGATTC  R: GCGCATGGTGACAAAGAGAGTGCAA | 881 | 64 |

Table S3 Primers of *wzi* loci of KP

| Capsular Serotyping | Primers（5’ →3’） | Product (bp) | Temperature (℃) |
| --- | --- | --- | --- |
| *wzi* | F：GTGCCGCGAGCGCTTTCTATCTTGGTATTCC  R：GAGAGCCACTGGTTCCAGAATTACCGC | 580 | 55 |

Table S4 Primers of housekeeping genes of KP

| Housekeeping genes | Primers（5’ → 3’） | Product (bp) |
| --- | --- | --- |
| *rpoB* | F：GTTTTCCCAGTCACGACGTTG AGGCGAAATGGCWGAGAACCA  R：TTGTGAGCGGATAACAATTTCGAGTCTTGGAAGTTGTAACC | 501 |
| *gapA* | F：GTTTTCCCAGTCACGACGTTGTATGAAATATGACTCCACTCACGG  R：TTGTGAGCGGATAACAATTTCCTTCAGAAGCGGCTTTGATGGCTT | 450 |
| *mdh* | F：GTTTTCCCAGTCACGACGTTGTACCCAACTCGCTTCAGGTTCAG  R：TTGTGAGCGGATAACAATTTCCCGTTTTTCCCCAGCAGCAG | 477 |
| *pgi* | F：GTTTTCCCAGTCACGACGTTGTAGAGAAAAACCTGCCTGTACTGC  R：TTGTGAGCGGATAACAATTTCCGCGCCACGCTTTATAGCGGTTAAT | 432 |
| *phoE* | F：GTTTTCCCAGTCACGACGTTGTAACCTACCGCAACACCGACTTCT  R：TTGTGAGCGGATAACAATTTCTGATCAGAACTGGTAGGTGAT | 420 |
| *infB* | F：GTTTTCCCAGTCACGACGTTGTACTCGCTGCTGGACTATATTCG  R：TTGTGAGCGGATAACAATTTCCGCTTTCAGCTCAAGAACTTC | 318 |
| *tonB* | F：GTTTTCCCAGTCACGACGTTGTACTTTATACCTCGGTACATCAGGT  R：TTGTGAGCGGATAACAATTTCATTCGCCGGCTGRGCRGAGAG | 414 |
